# A large-scale investigation into the role of classical HLA loci in multiple types of severe infections, with a focus on overlaps with autoimmune and mental disorders

**DOI:** 10.1186/s12967-021-02888-1

**Published:** 2021-05-31

**Authors:** Ron Nudel, Rosa Lundbye Allesøe, Wesley K. Thompson, Thomas Werge, Simon Rasmussen, Michael E. Benros

**Affiliations:** 1 Institute of Biological Psychiatry, Mental Health Centre Sct. Hans, Mental Health Services Copenhagen, Roskilde, Denmark; 2 iPSYCH, The Lundbeck Foundation Initiative for Integrative Psychiatric Research, Aarhus, Denmark; 3 CORE – Copenhagen Research Centre for Mental Health, Mental Health Centre Copenhagen, Copenhagen University Hospital, Copenhagen, Denmark; 4 Novo Nordisk Foundation Center for Protein Research, Faculty of Health and Medical Sciences, University of Copenhagen, Copenhagen, Denmark; 5 Division of Biostatistics, Herbert Wertheim School of Public Health and Human Longevity Science, University of California, San Diego, CA USA; 6 Department of Clinical Medicine, Faculty of Health and Medical Sciences, University of Copenhagen, Copenhagen, Denmark; 7 Department of Immunology and Microbiology, Faculty of Health and Medical Sciences, University of Copenhagen, Copenhagen, Denmark

**Keywords:** HLA, MHC, Association study, Immunogenetics, Infections, Autoimmune disease, Psychiatric disorder

## Abstract

**Background:**

Infections are a major disease burden worldwide. While they are caused by external pathogens, host genetics also plays a part in susceptibility to infections. Past studies have reported diverse associations between human leukocyte antigen (HLA) alleles and infections, but many were limited by small sample sizes and/or focused on only one infection.

**Methods:**

We performed an immunogenetic association study examining 13 categories of severe infection (bacterial, viral, central nervous system, gastrointestinal, genital, hepatitis, otitis, pregnancy-related, respiratory, sepsis, skin infection, urological and other infections), as well as a phenotype for having any infection, and seven classical HLA loci (HLA-*A, B, C, DPB1, DQA1, DQB1* and *DRB1*). Additionally, we examined associations between infections and specific alleles highlighted in our previous studies of psychiatric disorders and autoimmune disease, as these conditions are known to be linked to infections.

**Results:**

Associations between HLA loci and infections were generally not strong. Highlighted associations included associations between DQB1*0302 and DQB1*0604 and viral infections (P = 0.002835 and P = 0.014332, respectively), DQB1*0503 and sepsis (P = 0.006053), and DQA1*0301 with “other” infections (a category which includes infections not included in our main categories e.g. protozoan infections) (P = 0.000369). Some HLA alleles implicated in autoimmune diseases showed association with susceptibility to infections, but the latter associations were generally weaker, or with opposite trends (in the case of *HLA-C* alleles, but not with alleles of HLA class II genes). HLA alleles associated with psychiatric disorders did not show association with susceptibility to infections.

**Conclusions:**

Our results suggest that classical HLA alleles do not play a large role in the etiology of severe infections. The discordant association trends with autoimmune disease for some alleles could contribute to mechanistic theories of disease etiology.

**Supplementary Information:**

The online version contains supplementary material available at 10.1186/s12967-021-02888-1.

## Background

According to a World Health Organization report, infections are one of the major global burdens of disease [1], and they are a leading cause of early childhood mortality even today [2]. Not only can infections be deadly on their own, but they often exacerbate existing conditions, often leading to fatal consequences [3, 4]. Given the above, studying the genetic basis of susceptibility to infection is of major importance in order both to identify individuals at high risk and also to gain a better understanding of the infection mechanism. At the time of writing this article, the global community is experiencing a pandemic caused by SARS-CoV-2; genetic studies (including ones examining the genes included in this study) are already providing useful information in the battle against the virus, but, especially when polymorphic loci are involved, the results illustrate the need for large-scale studies [5].

Some of the most important genetic loci that are involved in immune reaction are the classical human leukocyte antigen (HLA) genes, found in the human major histocompatibility complex (MHC) region on chromosome 6. Genes of HLA classes I and II are involved in antigen presentation to T cells, whereby HLA class I genes encode proteins that present endogenous antigens to CD8^+^ T_C_ (cytotoxic T) cells and interact with natural killer (NK) cells, and HLA class II genes encode proteins that present exogenous antigens to CD4^+^ T_H_ (T helper) cells [6]. Furthermore, some HLA genes are extremely polymorophic [7]. These two aspects of HLA genes made them popular candidates for investigations into susceptibility to infections of various kinds and in various populations, resulting in many reported associations [8, 9]. However, as noted by other authors [8], many of the reported HLA associations suffer from publication bias, and the studies reporting them often had small sample sizes. In parallel with investigations of HLA genes in the context of infections, HLA genes have also been studied in the contexts of autoimmune diseases [10] and psychiatric disorders such as schizophrenia [11]. Interestingly, both susceptibility to infection and autoimmune disorders have been linked to psychiatric disorders, both genetically and from an epidemiological perspective [12–14]. Our own previous studies have also examined associations between classical HLA alleles and psychiatric disorders [15], as well as overall autoimmune disease [13].

In this study we test for association between HLA loci and susceptibility to severe infections utilizing a large, genetically homogeneous Danish sample from the iPSYCH2012 study, which included register-based diagnoses for psychiatric disorders, infections and autoimmune diseases as well as genetic data. The aim of this study was twofold: firstly, we wanted to test for genetic association between HLA alleles and multiple infection categories representing severe infections (infections requiring hospitalization). In this regard, our sample size, which included more than 10,000 cases for some infection categories e.g. bacterial or viral infections, is a vast improvement compared to most of the previous studies. Secondly, we wanted to examine specific alleles highlighted in our previous studies of psychiatric disorders and autoimmune disease (namely, B*5701, C*0202, C*0304, C*0401, C*0702, DPB1*0301, DPB1*0402, DPB1*1501, DQA1*0102, DQA1*0301, DQA1*0401, DQA1*0501, DQB1*0201, DQB1*0302, DQB1*0402, DQB1*0501, DQB1*0602, DRB1*0301, DRB1*0401, DRB1*0405, DRB1*0801 and DRB1*1501) to see what effects they had on susceptibility to severe infections.

## Methods

### Data sources for diagnoses and study sample

Data were obtained by linking Danish population-based registers using the unique personal identification number employed in Denmark since 1968 [16]. The Danish Neonatal Screening Biobank stores dried blood spots taken 4–7 days after birth from nearly all infants born in Denmark after 1981 [16, 17]. Information about infections was obtained from the Danish National Hospital Registry, which, since 1977, contains records of all inpatients treated in Danish non-psychiatric hospitals, and, since 1995, contains information regarding outpatient and emergency room contacts [18]. The Psychiatric Central Research Register covers all psychiatric inpatient facilities since 1969 and outpatient contacts since 1995 [19]. Diagnostic information was based on the 8th Revision of the International Classification of Diseases (ICD-8) [20] from 1977 to 1993, and ICD-10 from 1994 [21]. The individuals in this study are part of the iPSYCH 2012 cohort [22], nested within all individuals in the Danish population born between 1981 and 2005 (N = 1,472,762), and which included individuals diagnosed with at least one of: schizophrenia, bipolar disorder or depression (affective disorder), autism spectrum disorder, attention deficit/hyperactivity disorder and anorexia, and individuals included as part of a random population sample. Data pertaining to hospitalization for infections for all individuals in our study were obtained from the National Hospital Registry as described above. The iPSYCH sample has undergone extensive quality control (QC) as described in our previous studies which used imputed HLA alleles or infection diagnoses [12, 14, 15]. Importantly, individuals were removed based on ancestry (if they did not have Danish ancestry, as determined from registry data of family history and genetic principal component analyses) and relatedness (if they were first or second degree relatives of other individuals in the sample prioritizing first iPSYCH cases and then individuals with a higher genotype call rate). Individuals were also removed based on missingness (1%), abnormal heterozygosity, ambiguous sex (based on genetic markers), or if they were duplicates of other individuals. The first study employing this QC protocol has more information about the procedures [23]. Before QC, we had genotypes for 78,050 individuals. Following genetic and record-based QC, 65,534 unrelated Danish individuals were retained for downstream analyses (34,705 males and 30,829 females). Data for infections for each individual were up to the end of 2012, and the data for the psychiatric diagnoses were up to the end of 2013. The following infection categories were included in this study: bacterial, viral, central nervous system (CNS), gastrointestinal, genital, hepatitis, otitis, pregnancy-related (this was described somewhat confusingly in previous papers, but it refers to an infection present in the mother, who is in iPSYCH, while pregnant with or during delivery of the child, or immediately thereafter), respiratory, sepsis, skin infection, urological or other infections. ICD-8 and ICD-10 codes for these categories can be found in Additional file 1: Table S1.^1^ Individuals without any of these infection diagnoses were defined as controls, and individuals with at least one diagnosis were also defined as cases for the “any infection” phenotype. Sample sizes for all infection categories are shown in Table 1. There was a small number of people diagnosed with HIV/AIDS (ICD-8: 07983; ICD-10: B20, B21, B22, B23, B24; N = 16). This group was too small for our analyses, and they all had at least one other infection category. They were not excluded as such, but we did not analyze this infection group on its own (they were considered cases for the “any infection” phenotype, and they were excluded from being infection controls).

**Table 1:** Sample sizes of individuals with and without infections requiring hospital contacts among 65,534 individuals (45,889 with psychiatric diagnosis and 19,645 without one)

Group	Sample size
Cases	
Any infection	28,472
Type of infection	
Bacterial infections	11,833
Viral infections	11,914
Site of infection	
CNS infections	551
Gastrointestinal infections	7197
Genital infections	749
Hepatitis infections	111
Otitis media infections	5957
Pregnancy-related infections	661
Respiratory infections	12,958
Sepsis	438
Skin infections	4709
Urological infections	2106
Other infections	10,203
Controls	
Individuals without any infection	37,062

### Imputation of classical HLA alleles

Samples were genotyped on the Illumina Infinium PsychArray v1.0, as described in the original iPSYCH paper [22]. The dataset used to impute HLA alleles underwent QC as described in the original iPSYCH paper and a later iPSYCH study [47]. We were supplied with a dataset of 78,050 samples in 23 genotyping waves (this QC also applies to our first HLA study [15]). For the association analyses we used the final list of samples as per the procedure described in the previous section, meaning that samples not passing the QC described under "data sources for diagnoses and study sample" were excluded from downstream analyses; only 65,534 samples were used after the HLA imputation. As described previously [15], single-nucleotide polymorphism data were used to impute HLA types with a four-digit resolution for: *HLA-A, HLA-B, HLA-C, HLA-DRB1, HLA-DQA1, HLA-DQB1* and *HLA-DPB1*. The HLA imputation was performed with HIBAG [24] v1.3 using a pre-trained four-digit European ancestry model based on the PsychArray-B genotype platform (downloaded from: http://zhengxwen.github.io/HIBAG/hibag_index.html). The post-imputation QC included a posterior probability inclusion threshold of 0.9 for HLA alleles used in downstream analyses. In total, the following numbers of alleles were imputed for *HLA-A, HLA-B, HLA-C, HLA-DPB1, HLADQA1, HLA-DQB1,* and *HLA-DRB1*, respectively: 31, 65, 30, 21, 15, 17, and 43. After allele and sample QC, the following numbers of alleles remained: 24, 42, 21, 15, 12, 14, and 27. Our previous paper contains detailed statistical information about the imputed alleles and the quality of the imputation.

### Statistical analyses

As in our association analyses of HLA alleles and psychiatric [15] and autoimmune diseases [13], we employed a two-stage design. Gene-based tests were likelihood ratio tests for two logistic regression models run with the *glm* function in R [25] v3.3.1: (i) a full model, which included numeric variables for all HLA alleles for a given gene (with possible values of 0, 1 or 2, denoting the allele count per allele per individual) and covariates for age, age squared (to account for non-linearity with age), sex, the first ten principle components (to account for subtle differences in genetic ancestry) and having a psychiatric diagnosis (ICD-8: 290-315; ICD-10: F00-F99), and (ii) a null model, which included only the covariates (without the allele variables). The p-values are obtained from the chi-squared statistics using the *anova* function in R. The gene-based tests are omnibus tests which are meant to detect an overall association between an HLA gene and an infection category. It is not possible to make inferences about the effects of individual alleles from these models due to multicollinearity across allele variables. Furthermore, these tests may not work when very rare alleles are present or with small sample sizes/very few observations for cases, due to the influences of these factors on the regression in the full model. However, they can be used as a tool for assessing whether an association signal can be detected, as the full model as a whole may still be valid, as long as one does not try to determine the contributions of the individual independent variables from it [26]. Thus, these tests help focus downstream analyses on specific infection-gene pairs and reduce the overall number of tests. We did not consider infection-gene pairs for further analysis, if the regression/likelihood ratio test for them failed. In sum, these tests offer a tradeoff between a reduction in multiple testing and possibly missing individual allelic associations when the disease is rare or when there are rare occurrences of some alleles (however, the effect of an allele might not be estimated accurately even when tested alone, and the regression model for it might not work, if it has too few occurrences and/or the sample size for the specific regression is too small). Allele-based tests are post hoc tests which are employed to investigate the effects of specific alleles on the infection phenotype. They are logistic regressions of the infection status on the allele count of only one allele and the above covariates. These tests reveal the log-additive effects of specific HLA alleles on disease risk. The reported p-values for these tests are for whether the coefficient [log-odds ratio (OR)] for the allele count is different from zero (Wald Z test), as implemented in the *glm* function in R. False discovery rate (FDR) q-values were calculated using the QVALUE R package with the bootstrap method for all gene-based tests together and for the allele-based tests for each tested disease-gene pair both separately and across all tests, where possible (based on the p-value distributions); otherwise a lambda value of 0 was used [27].

### Comparison with autoimmune disease and mental illness and network analysis

We tested the top alleles associated with a psychiatric disorder or overall autoimmune disease from our previous studies in the context of association with infections. For associations with infections, we visualized the results of all allelic associations which had at least nominally significant p-values (P ≤ 0.05) with at least one infection category or with the “any infection” phenotype. The network was created with Cytoscape [28] v3.8.1. The color of the edges represents the direction of effect (red = risk; blue = protective), and the thickness of the edges corresponds to the absolute value of the estimate (ln(OR)) from the regression.

## Results

### Associations between HLA alleles and infections

Table 1 presents all infection categories included in our study and their corresponding sample sizes. In the HLA gene-based tests, 10 tests were nominally significant (P ≤ 0.05), as can be seen in Table 2. FDR analysis using all p-values obtained a minimum q-value of 27% for the associations with the four lowest p-values, namely *HLA-DQB1* with sepsis infection, *HLA-DQA1* with other infections, *HLA-DPB1* with CNS infection and *HLA-DQB1* and viral infection. A q-value of 27% for these four associations suggests a proportion of false positives of 27% among these i.e. about one of these four associations is expected to be a false positive, although there is no indication as to which one it is. We therefore tested all four with the allele-based tests. The results are presented in Table 3. The allele-based tests highlighted associations between DQB1*0503 and DQB1*0301 with sepsis, DQA1*0301 and DQA1*0103 with other infections and DQB1*0302 and DQB1*0604 with viral infections (with opposite trends in these cases). In terms of their q-values, when each gene-infection pair is tested alone, the associations between DQA1*0301 and other infections, and DQB1*0302 and DQB1*0604 and viral infections obtain q < 0.05. When tested together, only the associations between DQA1*0301 and other infections and DQB1*0302 and viral infections obtain q < 0.05. The association between DQB1*0503 and sepsis obtained q = 0.052 in this case. For certain viral infections, prior studies have shown that zygosity at HLA class II loci might have an effect on infection risk or on the severity of the infection [29–31]. We therefore tested whether there was an association between zygosity at class II loci and having a viral infection. We performed a two-sided Fisher’s exact test in R using a 2 × 2 table with counts of individuals (heterozygous/homozygous) and case/control status for viral infections. We did not find any significant associations for *DQB1*, *DRB1*, *DPB1* or *DQA1* (OR = 1.006, 0.967, 1.009, 0.994; P = 0.863, 0.417, 0.757, 0.844, respectively).

**Table 2:** Results of the gene-based likelihood ratio tests (p-values for each test are shown)

Disease/gene	HLA-A	HLA-B	HLA-C	HLA-DPB1	HLA-DQA1	HLA-DQB1	HLA-DRB1
Any infection	0.1759	0.6097	0.7975	0.6141	0.1708	0.5422	0.4631
Bacterial infection	0.3187	0.1235	0.05251	0.7455	0.7642	0.9248	0.8715
CNS infection	NA	NA	0.5737	0.01279^a^	0.6255	0.477	NA
Gastrointestinal infection	0.5783	0.5979	0.9051	0.03784	0.09455	0.2796	0.4751
Genital infection	1	1	0.3614	0.1255	0.4617	0.2426	NA
Hepatitis infection	NA	NA	NA	NA	0.07716	0.6711	NA
Other infections	0.643	0.6957	0.9214	0.7476	0.01073^a^	0.08866	0.4972
Otitis infection	0.02872	0.8145	0.6157	0.8382	0.8545	0.8985	0.5372
Pregnancy-related infection	0.6392	NA	0.7479	0.4424	0.8963	0.8691	NA
Respiratory infection	0.437	0.7647	0.2972	0.4032	0.1343	0.09267	0.1911
Sepsis	NA	NA	0.4429	0.7941	0.3766	0.003534^a^	NA
Skin infection	0.5236	0.02606	0.2706	0.7474	0.5656	0.6319	0.816
Urological infection	0.6288	0.7088	0.02561	0.3027	0.9862	0.6622	0.6907
Viral infection	0.2659	0.2998	0.3384	0.3027	0.03678	0.01402^a^	0.0467

NA denotes cases in which the regression/likelihood ratio test failed due to small sample sizes, multicollinearity of the alleles, and/or rare alleles

^a^These associations obtained the lowest q-value (27%)

**Table 3:** Results of the allele-based tests for top associations from the gene-based tests (only results for regressions that worked are shown; ORs were calculated before the truncation of some of the decimal places from the estimates)

Infection category	Allele	Estimate (ln(OR))	Std. error of the estimate	OR (odds ratio)	p-value
Sepsis	DQB1*0503	0.559104	0.203687	1.749105	0.006053
Sepsis	DQB1*0301	− 0.24618	0.105892	0.78178	0.020081
Sepsis	DQB1*0602	0.184527	0.095393	1.20265	0.053066
Sepsis	DQB1*0502	− 1.25217	0.710498	0.285883	0.078004
Sepsis	DQB1*0501	0.172764	0.10667	1.188585	0.105317
Sepsis	DQB1*0303	0.212767	0.156171	1.237096	0.173072
Sepsis	DQB1*0302	− 0.14869	0.112826	0.861833	0.187535
Sepsis	DQB1*0603	− 0.20829	0.159683	0.811969	0.192091
Sepsis	DQB1*0609	− 1.14838	1.003663	0.317152	0.252547
Sepsis	DQB1*0402	− 0.23573	0.212697	0.789997	0.267743
Sepsis	DQB1*0201	0.104055	0.126681	1.109661	0.411423
Sepsis	DQB1*0604	− 0.11078	0.161356	0.895133	0.492351
Sepsis	DQB1*0202	0.086373	0.132958	1.090213	0.515934
Sepsis	DQB1*0601	− 0.16911	0.506018	0.844415	0.738229
Other infections	DQA1*0301	0.094638	0.026576	1.099261	0.000369
Other infections	DQA1*0103	− 0.0716	0.03371	0.930902	0.033665
Other infections	DQA1*0601	− 0.46193	0.263865	0.630068	0.080012
Other infections	DQA1*0102	− 0.028	0.020206	0.972385	0.165775
Other infections	DQA1*0401	0.051297	0.045862	1.052635	0.263356
Other infections	DQA1*0303	0.040546	0.037877	1.041379	0.284414
Other infections	DQA1*0201	− 0.02777	0.027843	0.97261	0.318535
Other infections	DQA1*0101	− 0.02144	0.028011	0.978789	0.444044
Other infections	DQA1*0505	0.014933	0.028517	1.015045	0.600533
Other infections	DQA1*0104	0.035278	0.088494	1.035908	0.690147
Other infections	DQA1*0302	− 0.04039	0.117462	0.960416	0.730961
Other infections	DQA1*0501	0.000725	0.030631	1.000725	0.981128
CNS infection	DPB1*1101	− 0.77846	0.452205	0.459111	0.085163
CNS infection	DPB1*1401	1.210008	0.727933	3.353512	0.096462
CNS infection	DPB1*1001	− 0.76275	0.504907	0.46638	0.13087
CNS infection	DPB1*1301	− 0.66143	0.451917	0.516111	0.143299
CNS infection	DPB1*0201	− 0.16001	0.129761	0.852137	0.21754
CNS infection	DPB1*0101	0.179176	0.14896	1.196231	0.229037
CNS infection	DPB1*1601	− 0.61661	0.5786	0.539769	0.286559
CNS infection	DPB1*1501	− 0.6103	0.581432	0.54319	0.293882
CNS infection	DPB1*0301	0.106403	0.113766	1.11227	0.349642
CNS infection	DPB1*0402	0.096617	0.112958	1.101438	0.392365
CNS infection	DPB1*0401	0.053141	0.074281	1.054578	0.474359
CNS infection	DPB1*1901	− 0.25458	0.451412	0.775241	0.572777
CNS infection	DPB1*0501	0.06585	0.210093	1.068066	0.753953
CNS infection	DPB1*1701	0.025801	0.338575	1.026136	0.939257
CNS infection	DPB1*10401^a^	− 13.2288	210.2299	1.8E−06	0.949826
Viral infection	DQB1*0302	0.06831	0.022884	1.070697	0.002835
Viral infection	DQB1*0604	− 0.08484	0.034645	0.918659	0.014332
Viral infection	DQB1*0609	− 0.24632	0.13764	0.78167	0.073516
Viral infection	DQB1*0201	0.046721	0.027877	1.04783	0.093743
Viral infection	DQB1*0603	− 0.04453	0.032445	0.956449	0.169931
Viral infection	DQB1*0303	− 0.04964	0.038459	0.951569	0.196766
Viral infection	DQB1*0601	− 0.12332	0.107941	0.88398	0.253254
Viral infection	DQB1*0503	0.058851	0.057194	1.060617	0.303494
Viral infection	DQB1*0202	− 0.0249	0.030592	0.97541	0.41574
Viral infection	DQB1*0301	0.016236	0.021299	1.016369	0.445881
Viral infection	DQB1*0602	− 0.01546	0.022136	0.984661	0.484981
Viral infection	DQB1*0402	− 0.02045	0.04206	0.979754	0.62675
Viral infection	DQB1*0502	0.005057	0.085364	1.00507	0.95276
Viral infection	DQB1*0501	0.000455	0.024834	1.000455	0.985382

^a^The regression analysis for this allele resulted in a large standard error of its estimate due to its low frequency and therefore its effect cannot be determined accurately

### Comparison with associations of HLA alleles with autoimmune disease and mental illness

**Fig. 1:**
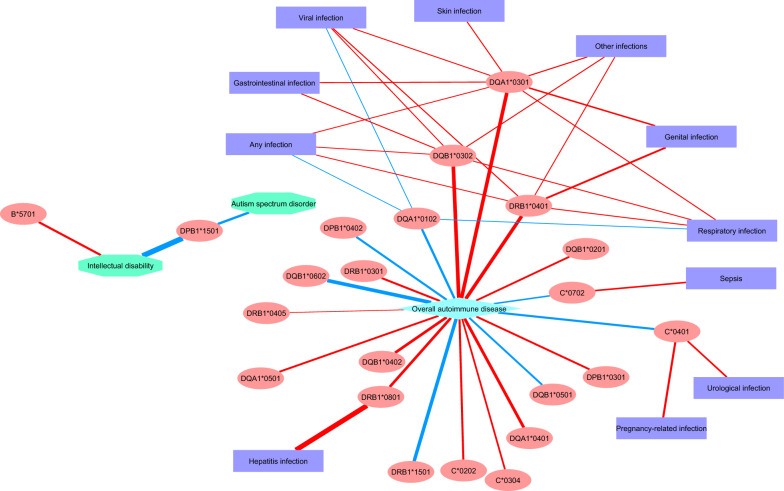
Network visualization of links between highlighted HLA alleles, mental illness, autoimmune disease and infection. The color of the edges represents the direction of effect (red = risk; blue = protective), and the thickness of the edges corresponds to the absolute value of the estimate (lnOR) from the regression

Twenty-two alleles were highlighted in our previous studies: 20 alleles for autoimmune disease and 2 alleles for mental illness (see “Background”). As both infection and autoimmune disease are correlated with mental illness, and as the immune system is intrinsically linked to both infection and autoimmune disease, we examined potential associations between the 22 alleles highlighted in our previous studies and all infection categories. We obtained 24 nominally significant associations with at least one infection category (Table 4). The results are also visualized as a network in Fig. 1. Two interesting points to note are the following: (i) while alleles implicated in autoimmune disease are well-connected to infections, alleles implicated in autism spectrum disorder and/or intellectual disability are not connected to infections at all; (ii) regarding alleles connected to both infections and autoimmune disease, with the exception of hepatitis, the effects of those alleles on autoimmune disease are larger than on infections, and the following pattern emerges: for HLA class I alleles (namely alleles of *HLA-C*), the trends are discordant between autoimmune disease and infection; they are protective for the former and increase the risk of the latter with similar effect sizes; for HLA class II alleles, the directions of effect are the same across both types of diseases, and they are almost always stronger for autoimmune disease.

**Table 4:** Associations between infections and alleles implicated in autoimmune disease or mental illness from our previous studies (only associations with P ≤ 0.05 are shown; ORs were calculated before the truncation of some of the decimal places from the estimates)

Allele	Estimate (ln(OR))	Std. error of the estimate	OR (odds ratio)	p-value	Infection category
DQA1*0102	− 0.0292	0.014231	0.971221	0.040168	Any infection
DQA1*0301	0.052435	0.019077	1.053834	0.005984	Any infection
DQB1*0302	0.04346	0.017379	1.044418	0.012395	Any infection
DRB1*0401	0.051723	0.023683	1.053084	0.028967	Any infection
DQA1*0301	0.08244	0.030735	1.085933	0.007311	Gastrointestinal infection
DQB1*0302	0.074499	0.028013	1.077345	0.007826	Gastrointestinal infection
DQA1*0301	0.168838	0.084957	1.183928	0.046885	Genital infection
DRB1*0401	0.230091	0.103525	1.258714	0.026246	Genital infection
DRB1*0801	0.806151	0.353065	2.239273	0.022413	Hepatitis infection
DQA1*0301	0.094638	0.026576	1.099261	0.000369	Other infections
DQB1*0302	0.077648	0.024273	1.080742	0.001379	Other infections
DRB1*0401	0.065745	0.033446	1.067954	0.049336	Other infections
C*0401	0.277345	0.101347	1.319621	0.006208	Pregnancy-related infection
DQA1*0102	− 0.0369	0.018438	0.963772	0.045357	Respiratory infection
DQA1*0301	0.074712	0.024401	1.077574	0.0022	Respiratory infection
DQB1*0302	0.063564	0.022285	1.065628	0.004341	Respiratory infection
DRB1*0401	0.067727	0.030469	1.070073	0.026227	Respiratory infection
C*0702	0.182894	0.089832	1.200687	0.041754	Sepsis
DQA1*0301	0.081349	0.036639	1.084749	0.026398	Skin infection
C*0401	0.193844	0.057718	1.213907	0.000784	Urological infection
DQA1*0102	− 0.03836	0.019028	0.962365	0.0438	Viral infection
DQA1*0301	0.066253	0.025166	1.068497	0.008472	Viral infection
DQB1*0302	0.06831	0.022884	1.070697	0.002835	Viral infection
DRB1*0401	0.089667	0.031349	1.09381	0.004232	Viral infection

## Discussion

This paper reports a comprehensive immunogenetic association study of multiple categories of infections requiring hospitalization. Our sample sizes ranged from 111 cases for hepatitis to 28,472 cases for any severe infection (Table 1), making our study one of the largest genetic studies of infections to date.

We did not detect very strong associations between specific HLA loci and infections at the gene level. This is in sharp contrast to our previous findings with regards to autoimmune disease and, to a lesser extent, psychiatric disorders. This could be due to the intrinsic nature of infections, which are passed horizontally from individual to individual, making the study design less “controlled”. In this context it is also important to keep in mind the relatively low heritability for overall infection observed in our previous study using this cohort [14]. Alternatively, this could be the result of a small sample size for some infection categories combined with the allele frequencies of some rare alleles, or it could be due to the degree of heterogeneity of the infection phenotypes. For some viral infections, especially hepatitis B and C, the zygosity at specific HLA class II loci might also be important [29–31], but in some cases it pertained mostly to the severity of the infection. We did not observe this effect for class II loci in our study. With regards to differences in the results between our study and previous studies, they could also have arisen due to differences in the resolution of HLA typing, differences in the definitions of the phenotypes and/or population effects, and it should be noted that many of the old studies had very small samples [8]. Our results could also reflect true small effects. In this context it is important to mention a study by Tian et al. from 2017, which reported highly significant associations between HLA alleles and several specific infections [32]. However, this study examined specific common infections, whereas our study examined severe infections requiring hospitalization, in broader categories of infection type; this could also suggest different effects to risk of infection and severity of infection (although our current study cannot address this possibility). Moreover, the sample size of the 2017 study was over 200,000, thus allowing the detection of smaller effects. Most of the associations the authors report have, in fact, small to moderate effect sizes, albeit highly significant ones. Our own analyses nonetheless highlighted several classical HLA alleles. Several of these have been implicated in past studies of infections. A haplotype with DQB1*0503, which in our study was a risk allele for sepsis, had also been associated with severe systemic disease (SSD) in the absence of necrotizing fasciitis (NF) in the context of severe, invasive, group A streptococcal infection (GAS) [33]. In the same study, DQB1*0301, which reduced the risk of sepsis in our study, was associated with NF in the absence of SSD. While this study suggested interactions between these alleles, SSD and NF (in the context of GAS), our results demonstrate that what could be a risk allele for one complication could be protective for another; however, this is only speculative, as we did not investigate specific complications of infection. Nonetheless, as these two complications can be seen as either an over-reaction (SSD) or insufficient response (NF) of the immune system, these opposite effects do make some biological sense. DQA1*0301 (risk) and DQA1*0103 (protective) were associated with the “other infections” category. This infection category encompasses potentially very different infection diagnoses by definition, and so it may be hard to draw conclusions about these associations. However, these alleles were highlighted in past studies of gastrointestinal diseases or liver diseases: DQA1*0301 was reported as a risk factor for *Helicobacter pylori* infection [34]. Conversely, DQA1*03 was found to have a protective effect on chronic hepatitis C infection [35]. DQA1*0103, which was protective in our study, was found to be associated with spontaneous recovery from hepatitis B infection [36]. Lastly, DQB1*0302 (risk) and DQB1*0604 (protective) showed association with viral infections. Like DQA1*03, DQB1*0302 was found to reduce risk of chronic hepatitis C infection in the above study [35]. A haplotype with DQB1*0604 was associated with low hepatitis activity in the context of chronic hepatitis C infections [37].

In the second part of our study, we examined whether alleles previously associated with psychiatric disorders or autoimmune disease from our previous studies showed association with infections. As can be seen in Fig. 1, there were no common alleles to both psychiatric disorders and infections. In contrast, several of the alleles significantly associated with autoimmune disease showed some association with infections. With one notable exception, when an allele was associated in the same direction with both autoimmune disease and an infection, its effect was larger on the former. For *HLA-C* alleles showing association with both disease classes, the direction of association was discordant between autoimmune disease (protective) and infections (risk). The latter result could potentially be explained by considering a mechanism whereby some *HLA-C* alleles lead to low immune reactivity to specific ligands, thus lowering the risk of autoimmune disease but increasing the risk of infection, if there is some e.g. structural connection between an infectious antigen and a self-antigen the HLA molecule can bind. Some alleles are also known to have lower surface expression and other alternative expression patterns in general. A mechanism for a related scenario, whereby the binding capabilities of specific HLA molecules to self-antigens which resemble microbial peptides can lead to autoimmune disease, has been proposed, but there is conflicting evidence in this regard, and, in that scenario, the HLA molecule in question also showed extracellular binding capabilities [38–41]. Interestingly, a recent study reported cross-reactivity between an enterococcal bacteriophage peptide and tumor antigens binding to MHC class I molecules [42]. The associations with concordant trends across infections and autoimmune disease are conceptually harder to speculate about, perhaps, but it should be noted that, with the exception of DRB1*0801 and hepatitis, the effect sizes of the associations with infections in those cases are smaller, with the average absolute value of the effect size (regression coefficient) being ~ 0.08 (compared to ~ 0.47 for autoimmune disease). As noted above, one exception to this is the association between DRB1*0801 and hepatitis, which is stronger than the former’s association with autoimmune disease, and in the same direction. This allele, however, is consistently reported as associated with an autoimmune disease of the liver, namely, primary biliary cirrhosis (PBC) [43, 44]. Moreover, a diagnosis of PBC may be delayed in individuals with viral hepatitis [45], and a differential diagnosis between PBC and viral hepatitis can be difficult due to some PBC pathophysiology which can mimic chronic hepatitis, especially hepatitis C [46]. Since we do not have access to this kind of data for the individuals in our study, we cannot rule out that these factors could have potentially influenced the diagnosis and hence the observed association. Hepatitis was also the smallest infection category in our study in terms of sample size, which could suggest that the effect size for its association is inflated.

## Conclusions

In conclusion, while our study confirmed some previously reported associations with classical HLA alleles, the overall picture suggests that the effects of HLA alleles on susceptibility to severe infections are not large, especially when compared with their effects on risk of autoimmune disease. Some alleles, notably two *HLA-C* alleles, had discordant effects on susceptibility to infection and autoimmune disease, in line with some hypotheses regarding the origins of some autoimmune diseases. Unlike in the case of autoimmune disease, classical HLA alleles might not play a large role in the etiology of severe infections, although there is some evidence for their involvement therein.

## Supplementary Information

**Additional file 1: Table S1**. ICD-8 and ICD-10 codes for site and type of infection.

## Data Availability

iPSYCH data are stored in a national HPC facility in Denmark. The iPSYCH initiative is committed to providing access to these data to the scientific community, in accordance with Danish law. Researchers may be granted access upon request to the iPSYCH management.

## Abbreviations

CNS: Central nervous system
FDR: False discovery rate
GAS: Group A streptococcal infection
HLA: Human leukocyte antigen
ICD: International Classification of Diseases
MHC: Human major histocompatibility complex
NF: Necrotizing fasciitis
PBC: Primary biliary cirrhosis
QC: Quality control
SSD: Severe systemic disease

## Footnotes

1 This table corrects a few minor errors in the codes mainly for the bacterial and viral infection categories, as found in previous versions of it, and has more specific (higher resolution) codes for some infections.
