# Supplementary material for: A large-scale investigation into the role of classical HLA loci in multiple types of severe infections, with a focus on overlaps with autoimmune and mental disorders
Source: J Transl Med. 2021 May 31;19:230. doi: 10.1186/s12967-021-02888-1 (PMC8165335; doi:10.1186/s12967-021-02888-1)
Supplement: Supplementary file 1 — Additional file 1: Table S1. ICD-8 and ICD-10 codes for site and type of infection. [file 12967_2021_2888_MOESM1_ESM.docx]

| **Online Table 1: ICD-8 and ICD-10 codes for site and type of infection** | | |
| --- | --- | --- |
| Infection category | ICD-8 Codes | ICD-10 Codes |
| **Site of infection** | | |
| Sepsis infections | 038 | A40-A41 |
| Hepatitis infections | 070 | B15-B19, K770A, K770B |
| Gastrointestinal infections | 000-009, 540 | A00-A09, K35 |
| Skin infection | 035, 050-057, 110-111, 680-686 | A46, B00-B09, L00-L08 |
| Respiratory infections | 460-486 | J00-J18, J22, J36 |
| Urological infections | 580, 590, 595 | N00, N05, N129, N300, N370, N390 |
| Genital infection | 604, 612, 620, 622 | N45, N512, N70, N700, N709, N760, N762, N764, N770, N771, N518B |
| Pregnancy-related infections | 630, 635, 670 | O23, O264, O85-O86, O98 |
| Otitis media infections | 381-382 | H65-H67 |
| Central nervous system infections | 013, 02701, 03609, 040-043, 04509, 04519, 04599, 046, 05201, 05302, 05403, 05501, 05601, 06209, 06219, 06229, 06239, 06249, 06299, 06309, 06319, 06329, 06399, 06499, 06599, 07199, 07202, 07501, 07929, 09049, 09490, 0949, 320, 322-324, 474 | A066, A022C, A17, A229C, A321, A390, A504, A514B, A521, A521A, A521B, A548A, A548D, A80-A89, B003, B004, B010, B011, B020, B021, B050, B051, B060, B261, B262, B375, B451, B582, B602, E236A, G00-G07 |
| HIV/AIDS | 07983 | B20-B24 |
| **Type of infection** | | |
| Bacterial infections | 000, 00009, 00019, 00099, 00199, 002-005, 008, 01099, 011-018, 020-023, 025-027, 030-039, 07399, 07699, 07984, 08899, 08900, 08099, 081-083, 09009, 09049, 09059, 091-098, 100-104, 32009, 32019, 32080, 322, 380-382, 390-392, 420-421, 461-463, 46403, 481-483, 50199, 50800, 50801, 50802, 50803, 510, 513, 52259, 52649, 52722, 52838, 52839, 52903, 54001, 566-567, 57703, 590, 59500, 59501, 59700, 59703, 59900, 59906, 601, 604, 60739, 61100, 61101, 612, 614, 62090, 62099, 622, 62949, 630, 63109, 63110, 63111, 63119, 63129, 63139, 635, 670, 67801, 680, 68108, 68109, 682, 68399, 68408, 68409, 68501, 68600, 68608, 710, 7200, 7201, 7202, 72031, 73299 | A022C, A03-A05, A15-A28, A30-A58, A65-A79, B95-B96, B088D, D733, E060A, E236A, E321, I301A, I301B, I301C, I301D, I320, I410, I430, I520, J01, J020, J030, J13-J15, J160, J170, J851, J86, J860, J869, K040A, K046A, K052A, K112A, K130A, K140A, K209A, K113, K122, K351, K61, K650N, K67, K630, K930, L00- L04, L08, M00, M010-M013, M015B, M014, M463, M490-M492, M680, M725A, N10, N12, N136, N151, N200I, N201I, N300, N309, N340, N341, N390, N410, N412, N431, N450, N459, N510A, N510C, N511, N70, N71, N72, N73, N74, N740, N741, N742, N743, N744, N764, N980, O23, O753, O85-O86, O980, O981, O982, T793, T802, T814, T874, T880 |
| Viral infections | 00880, 00889, 00890, 040-046, 050-057, 060-065, 067-068, 070-072, 074-075, 46099, 464, 46599, 47099, 471-474, 48099 | A08, A60, A630, A80-A89, A90-A99, B00, B060, B01-B09, B15-B26, B260, B261, B262, B263, B268, B269, B27, B270, B271, B278, B279, B33, B330, B331, B332, B333, B338, B34, B340, B341, B342, B343, B344, B348, B349, B97, G020, G051, H621A, H621B, H671A, H671B, I301E, I400B, I411A, I411B, J00, J04-J06, J10- J12, J050, J171, J203, J204, J205, J206, J207, J210, K770A, K770B, K871A, K871B, M014, M015, M015A, N518B, N770D, N771B, N771G, N771L |
| Other infections | 006-007, 00899, 00999, 084, 087, 089, 09991, 09992, 09993, 09999, 110-117, 120-131, 13603, 13600, 572, 99859 | A06-A07, A085, A09, A59, A63-A64, B35-B60, B64-B83, B87-B89, B99, G02, G040, G049, G052, G079D, H622, I301, I400, I411, I412, I521C, I33, J18, J02-J03, J172-J173, J178, J22, J998B, J998C, K770C, K770D, K770E, K35, K750, L303, M016, M631C, M631D, M631E, M631F, M632A, M651, M711, N370A, N160D, O983, O986-O989, T89 |

Notes for this table: we have amended this table to reflect the exact codes that were extracted from the register, as we discovered that the previous versions ([1](#_ENREF_1), [2](#_ENREF_2)) had some minor errors/omissions. This involved 4 codes that were in the original table but not included in the study (in the viral or bacterial infection categories, namely I398, N11, 078 and 07983, the latter of which was mistakenly written as 079.82), and 4 codes that were included but were not noted in original the table (also in the viral or bacterial infection categories, namely N309, N340, I301E and J050). Regarding the former group, however, the frequency of these codes among individuals born between 1981 and 2005 (same year of birth range as in iPSYCH) in the entire Danish population (from hospital registry data until 2017) are 0.000009, 0.00039, 0.000019 and 0.0000009, respectively. Therefore, we do not expect to have many individuals in iPSYCH who had only these codes and none of the other ones, who were consequently erroneously treated as controls. Also, not all codes are, in fact, infection diagnoses per se (I398, N11).

Additionally, we provide a higher resolution (more specific) code for some diagnoses; in most cases this does not reflect any change e.g. where the previous version listed ICD-8 code “062” and this version lists “06209, 06219, 06229, 06239, 06249, 06299”, and only these six codes appear in the Danish ICD-8 under 062. Cases in which ICD-8 had more codes than listed here include 089 and 599, but overall these were very rare, with frequencies of 0.000002 and 0.00004, respectively, for the higher resolution codes that were not included in the study. When a lower resolution code is listed in the table, it can be understood as including all diagnoses under its ICD entry. For example, ICD-8 060 includes 06009, 06019 etc.

As the two previous studies examined either only gastrointestinal infections (no errors found) or any infection (all codes regardless of category), and the erroneously excluded diagnoses were very rare, this did not have a major effect on our previous studies, but, since this study examined at all individual infection categories, we provide this updated table.

1. Nudel R, Appadurai V, Schork AJ, Buil A, Bybjerg-Grauholm J, Borglum AD, et al. A large population-based investigation into the genetics of susceptibility to gastrointestinal infections and the link between gastrointestinal infections and mental illness. Human genetics. 2020;139(5):593-604. Epub 2020/03/11.

2. Nudel R, Wang Y, Appadurai V, Schork AJ, Buil A, Agerbo E, et al. A large-scale genomic investigation of susceptibility to infection and its association with mental disorders in the Danish population. Translational psychiatry. 2019;9(1):283. Epub 2019/11/13.
